# 2824. 3rd Generation Cephalosporin (3rd CEP) Use and Antibiotic Selection Appropriateness for Uncomplicated Febrile Urinary Tract Infections (UTI) in Children

**DOI:** 10.1093/ofid/ofad500.2435

**Published:** 2023-11-27

**Authors:** Marina Dantas, Allison Ross Eckard, Daniel C Williams, Taylor Morrisette, Stephen A Thacker, Natalie Pudalov, Ronald J Teufel

**Affiliations:** MUSC, Charleston, South Carolina; Medical University of South Carolina, Charleston, South Carolina; MUSC, Charleston, South Carolina; Medical University of South Carolina College of Pharmacy, Charleston, South Carolina; Medical University of South Carolina, Charleston, South Carolina; Medical University of South Carolina, Charleston, South Carolina; Medical University of South Carolina, Charleston, South Carolina

## Abstract

**Background:**

Concerns regarding antibiotic resistance have led many pediatricians to rely on 3^rd^ CEP, namely ceftriaxone (CRO) and cefdinir (CDR), for UTI treatment. 3^rd^ CEP use is a major risk factor for *C. difficile* infections and contribute to multidrug resistance. We sought to examine local prescribing patterns for uncomplicated febrile UTI in children to guide development of a standardized treatment pathway aimed at reducing unnecessary 3^rd^ CEP use.

**Methods:**

We conducted chart review of children aged 2 mo.-18 yrs treated at our institution for uncomplicated febrile UTI between Oct 2021 – Oct 2022. Subjects were identified by abnormal urine culture (UCx) regardless of colony-forming units (CFU) and included if they met *a priori* definition. Antibiotic selections were evaluated relative to urinalysis (UA), UCx, and bacterial susceptibilities to assess appropriateness of treatment. This project was certified by our institutional review board as quality improvement.

**Results:**

61 subjects were included (85% female; 3.8 yrs median age; 70%, 16%, 13% treated in ED only, ED/inpatient, inpatient only, respectively). CRO or CDR were most frequently given as initial in-house treatment (ED or inpatient) (Fig 1). 53% of subjects received ≥1 dose of CRO or CDR (ED, inpatient or discharge prescription). 31% of subjects (68% in ED only) were identified as misdiagnosed with UTI (e.g., bacterial growth below CFU threshold or suggestive of contaminant). Providers relied on urine test strips rather than UA/microscopy in 38% of cases misdiagnosed UTIs. Of those with confirmed UTI, *E. coli* was most common pathogen (81%) followed by other Gram-negative bacteria (14%); susceptibilities were reported in 97% (Fig 2). Initial antibiotic selection was deemed inappropriate in 68% (Fig 3); 39% received CRO/CDR unnecessarily. ≥5 white blood cells per high-power field was most associated with a confirmed UTI (P=0.004).

**Figure:**
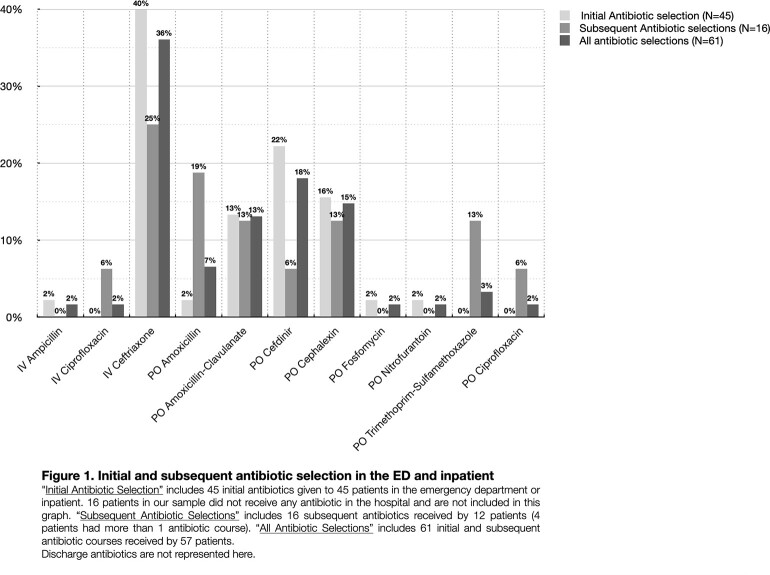

**Figure:**
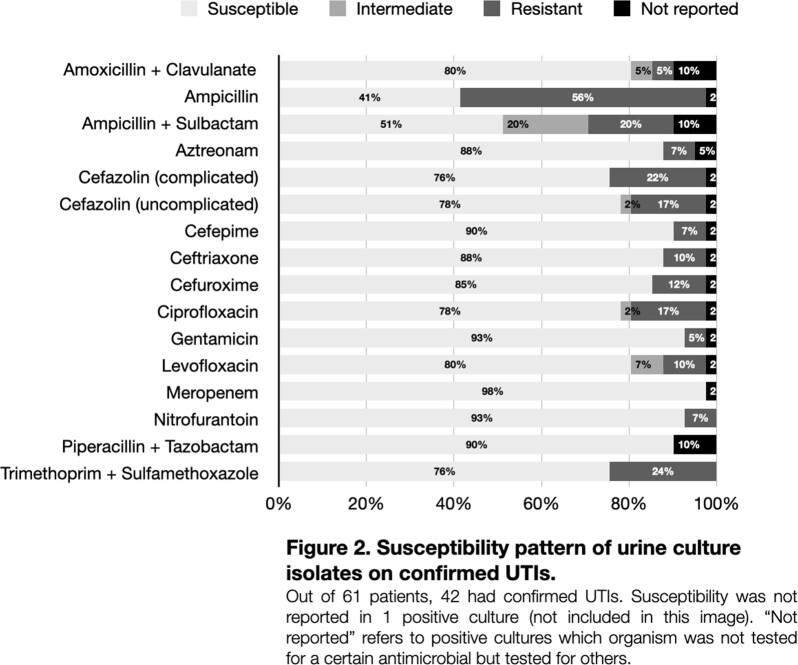

**Figure:**
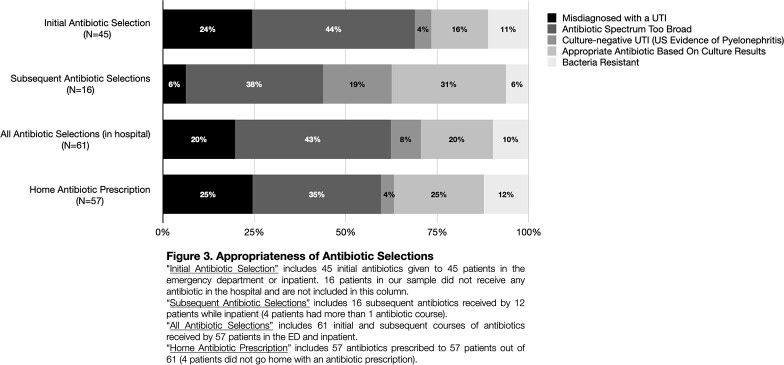

**Conclusion:**

Inappropriate 3^rd^ CEP use and antibiotic overuse in general were prevalent in our study. The biggest drivers were UA underutilization contributing to UTI misdiagnosis and failure to use UCx to de-escalate antibiotics. Our findings serve as a foundation from which to build a standardized treatment pathway for uncomplicated febrile UTIs that will improve antibiotic stewardship and reduce 3^rd^ CEP use.

**Disclosures:**

**Taylor Morrisette, PharmD, MPH**, AbbVie: Advisor/Consultant|Basilea: Advisor/Consultant **Ronald J. Teufel, II, MD, MSCR**, Enanta Pharmaceaticals: Grant/Research Support|Health Resources and Servises Administration: Grant/Research Support|Moderna: Grant/Research Support|National Institute of Health: Grant/Research Support

